# Editorial: Veterinary Bacterial Zoonoses

**DOI:** 10.3389/fvets.2018.00162

**Published:** 2018-08-06

**Authors:** Jiabo Ding, Menachem Banai, Shengqing Yu, Xin Ting

**Affiliations:** ^1^Department of Diagnostic Technology, China Institute of Veterinary Drug Control, Beijing, China; ^2^Department of Bacteriology, Kimron Veterinary Institute, Bet Dagan, Israel; ^3^Shanghai Veterinary Research Institute, Chinese Academy of Agricultural Sciences, Shanghai, China; ^4^Institute of Animal Sciences, Chinese Academy of Agricultural Sciences, Beijing, China

**Keywords:** bacterial zoonoses, bacterial bioinformatics, brucellosis, tuberculosis, paratuberculosis, chlamydia

Zoonotic diseases provide a unique angle of looking at the One Health attitude toward infectious diseases. One Health is a recent approach taken by the community in addressing different environmental risks, depending on the pathogenicity (disease manifestation by a bacterium in a host) and the way the disease is disseminated in the human environment. Here, zoonotic diseases add unique perspectives to the pathogens harnessing their animal hosts to spread in nature via their milk, placenta, and visceral organs, respectively, adding zoonotic risks to food borne diseases involving unpasteurized or uncooked materials.

In this Research Topic we chose *Brucella, Mycobacterium*, and *Chlamydia* as disease types which represent facultative vs. obligate intracellular parasites of eukaryotic cells, respectively. The facultative bacteria, although replicating as intracellular parasites, spread due to their ability to exist outside their hosts. *Chlamydia*, on the other hand, survive in the eukaryotic cells. *Brucella* exploit throphoblasts as a resource of cell replication until a critical cell mass can burst by induction of abortion, spreading the bacteria in the closest environment and sustaining the infection in naïve populations, respectively. *Mycobacterium* exploit a similar mechanism of developing a massive bacterial inoculum as an infective dose that spreads by aerosols. *Chlamydia*, as obligate intracellular parasites, can only establish a successful passage following direct contact between their hosts. Understanding these principles in disease dissemination thus provide different leads to controlling the disease by developing relevant scientific tools.

The zoonotic impact of a disease could be determined by studying the correlation between their common existence between animals and humans. High throughput serological tests have been established to conduct rapid and accurate mass testing of a population but these only provide estimates of the herd status without informing on the Brucella agent that was involved in the infection. For the latter, strain isolation and typing is needed. Similarly, serological tests cannot reveal the persistence of a pathogen in the animal population in comparison to its spread in the human population.

Critically, vaccines are used as an effective prophylactic means against diseases. However, should vaccines be developed to protect the animals or humans, even if doing so may have a negative economic effect on farmers? In cases where a vaccine has been successfully developed, how can we use it in the field without hampering serological diagnosis? Some of these questions are addressed in our Research Topic.

Dongri et al. used Multiple Locus-Variable Number Tandem Repeat Analysis (MLVA) as a means of analyzing the common existence of *B. canis* between canine and humans, proven to be highly specific in determining the epidemiology of the disease in the area.

Unlike *B. canis, B. melitensis* is considered the severest human pathogen associated with small ruminants. Similar to the case in Israel, *B. melitensis* is also endemic to China, and vaccination is included in both countries as a prophylactic measure aimed at reducing the spread of the disease. The Israeli laboratory is serving as a national and international reference laboratory for the disease. Because Rev. 1 vaccination is enforced in the country, here, Banai et al. have exploited their national position in developing DIVA tests (distinguishing infected from vaccinated animals). Therefore, a combined approach of screening the population using highly sensitive tests, such as Fluorescence polarization assay (FPA), and confirming the infection status, using the Complement Fixation Test (CFT), has shown promise in successfully determining the brucellosis cases in the small ruminants.

Sun et al. have characterized *B. melitensis* isolates in China using a combined strain typing approach that included both Multilocus Variable-number tandem-repeat Analysis (MLVA), Single Nucleotide Polymorphism (SNP), and Multilocus Sequence BruMLS. This showed that Chinese isolates are more closely related to isolates from Eastern Mediterranean and Middle Eastern countries.

Zhu et al. reported on the isolation of a novel, highly pathogenic field isolate of *M. avium* spp. Avium. This highlights the importance of bacteriological culturing of infected materials in showing possible existence of zoonotic variants within *M. avium*. Once an isolate has been obtained, data can be added to its genome. Recruiting strain isolation was also explored by Qi et al. as a method of studying antibiotic susceptibilities by an atypical enteropathogenic *E. coli* (EPEC) strain which added important information to control the disease in endangered Golden Snub-Nosed monkeys.

Due to a shortage in commercially available ELISA based detection kits, Xu et al. exploited the usage of mAbs targeting Ag85A from *M. tuberculosis* in surveying bovine tuberculosis. Developing monoclonal antibodies is a well-known approach in targeting specific epitopes. From five hybridomas two were selectively effective in distinguishing this antigen from Ag85B and At85C, allowing the pursuit of ELISA based detection of bovine tuberculosis.

*Chlamydia psittaci* causes chlamydiosis among birds which might then lead to avian influenza-like diseases in humans. Liu et al. compared the expression of PmpD-N of *C. psittaci* in the host, based upon two different promoters which contribute to the development of DNA vaccines against *C. psittaci*.

Research and development establish key components in advancing control and diagnosis of livestock diseases. As an example, Xin et al. explored means of detection of bovine tuberculosis based upon measurement of expression of IFN-⋎ and other cytokine mRNAs or proteins following induction of peripheral blood mononuclear cells from naturally or experimentally infected cattle by purified protein derivative (PPD-6) and mycobacterial early secretory ESAT-6 and culture filtrate CFP-10 proteins. Although showing signals that could be correlated with infection they concluded the test is still premature for field diagnosis.

A similar approach was undertaken by Zhang et al. in pursuit of the activation of the mitogen activated protein kinase (MAPK) signal by *B. abortus* outer membrane protein 25. For this, signals of the MAPK pathways were detected, such as p38 phosphorylation, and the extracellular-regulated protein kinase (ERK) and Jun-N-terminal kinase (JNK), were activated respectively. From this, the authors concluded that Omp25 played a role in *Brucella* activation of the MAPK signal pathway.

microRNAs (miRNAs) are well studied factors which function at the posttranscriptional step as regulators of biological pathways. Intriguingly, these molecules could be considered valuable in the diagnosis of infectious diseases, or as therapeutic agents, respectively. Dong et al. took the task of reviewing this subject in focusing on the possible exploitation of using miRNAs as biomarkers of infectious diseases among livestock.

To summarize, this Research Topic focuses on three important zoonotic diseases which explore bacterial diagnosis, epidemiology, laboratory works with clinical samples and research, and the development involved in advancing diagnostics as well as developing novel vaccines. We hope our Research Topic contributes to furthering the One Health approach.

## Author contributions

All authors listed have made a substantial, direct and intellectual contribution to the work, and approved it for publication.

### Conflict of interest statement

The authors declare that the research was conducted in the absence of any commercial or financial relationships that could be construed as a potential conflict of interest. The reviewer CS and handling Editor declared their shared affiliation.

